# Supplementary material for: Effects of Trichoderma harzianum combined with Phanerochaete chrysosporium on lignin degradation and humification during chicken manure and rice husk composting
Source: Front Microbiol. 2025 Feb 28;16:1515931. doi: 10.3389/fmicb.2025.1515931 (PMC11906335; doi:10.3389/fmicb.2025.1515931)
Supplement: Supplementary file 1 [file Data_Sheet_1.zip › 6.Function/All/FunGuild/l2/Freq.0.01.barplot_group.pdf]

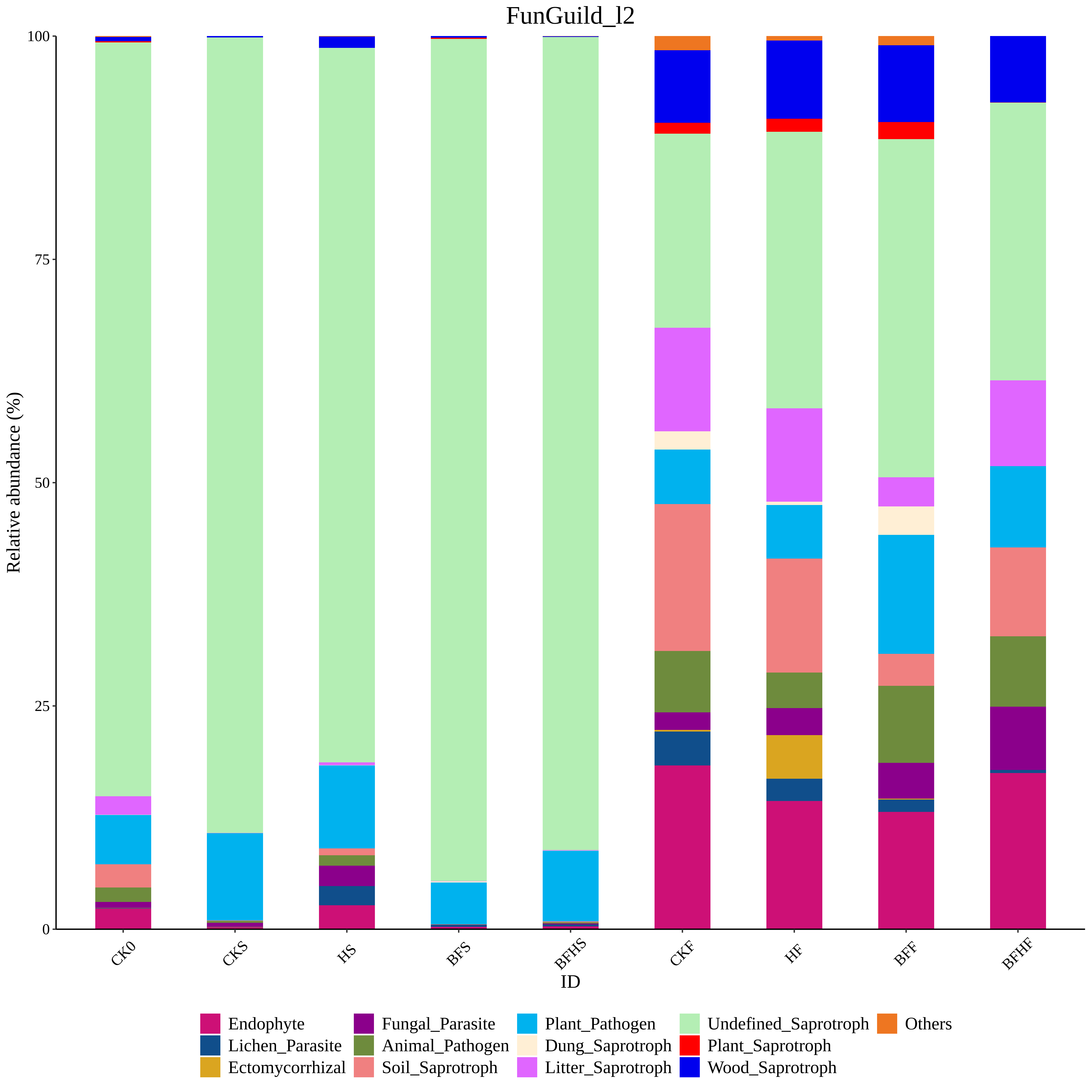

FunGuild_l2
Relative abundance (%)
100
75
50
25
0
CK0
CKS
HS
BFS
BFHS
CKF
HF
BFF
BFHF
ID
Endophyte
Lichen_Parasite
Ectomycorrhizal
Fungal_Parasite
Animal_Pathogen
Soil_Saprotroph
Plant_Pathogen
Dung_Saprotroph
Litter_Saprotroph
Undefined_Saprotroph
Plant_Saprotroph
Wood_Saprotroph
Others
